# The LCHADD Mouse Model Recapitulates Early-Stage Chorioretinopathy in LCHADD Patients

**DOI:** 10.1167/iovs.65.6.33

**Published:** 2024-06-21

**Authors:** Shannon J. Babcock, Allison G. Curtis, Garen Gaston, Gabriela Elizondo, Melanie B. Gillingham, Renee C. Ryals

**Affiliations:** 1 Department of Molecular and Medical Genetics, Oregon Health & Science University, Portland, Oregon, United States; 2 Casey Eye Institute, Oregon Health & Science University, Portland, Oregon, United States

**Keywords:** long-chain 3-hydroxyacyl-CoA dehydrogenase deficiency (LCHADD), chorioretinopathy, retinal pigment epithelium (RPE), mouse model

## Abstract

**Purpose:**

Recent studies have shown that the retinal pigment epithelium (RPE) relies on fatty acid oxidation (FAO) for energy, however, its role in overall retinal health is unknown. The only FAO disorder that presents with chorioretinopathy is long-chain 3-hydroxyacyl-CoA dehydrogenase deficiency (LCHADD). Studying the molecular mechanisms can lead to new treatments for patients and elucidate the role of FAO in the RPE. This paper characterizes the chorioretinopathy progression in a recently reported LCHADD mouse model.

**Methods:**

Visual assessments, such as optokinetic tracking and fundus imaging, were performed in wildtype (WT) and LCHADD mice at 3, 6, 10, and 12 months of age. Retinal morphology was analyzed in 12-month retinal cross-sections using hematoxylin and eosin (H&E), RPE65, CD68, and TUNEL staining, whereas RPE structure was assessed using transmission electron microscopy (TEM). Acylcarnitine profiles were measured in isolated RPE/sclera samples to determine if FAO was blocked. Bulk RNA-sequencing of 12 month old male WT mice and LCHADD RPE/sclera samples assessed gene expression changes.

**Results:**

LCHADD RPE/sclera samples had a 5- to 7-fold increase in long-chain hydroxyacylcarnitines compared to WT, suggesting an impaired LCHAD step in long-chain FAO. LCHADD mice have progressively decreased visual performance and increased RPE degeneration starting at 6 months. LCHADD RPE have an altered structure and a two-fold increase in macrophages in the subretinal space. Finally, LCHADD RPE/sclera have differentially expressed genes compared to WT, including downregulation of genes important for RPE function and angiogenesis.

**Conclusions:**

Overall, this LCHADD mouse model recapitulates early-stage chorioretinopathy seen in patients with LCHADD and is a useful model for studying LCHADD chorioretinopathy.

The retinal pigment epithelium (RPE) is the outermost retinal cell layer and is crucial for vision. Its ability to regulate the transport of nutrients and waste, to convert and store retinal pigments, and to phagocytose shed photoreceptor outer segments are all crucial in maintaining the function and health of the photoreceptors and choroid. Recent research has suggested that fatty acid β-oxidation (FAO) in the RPE is crucial in maintaining the metabolic homeostasis of the retina.^1^ Specifically, the RPE uses FAO to degrade fatty acids from shed outer segments, create ATP, and suppress RPE glycolysis so that glucose can pass from the choroid to the glycolytic photoreceptors.^2^^–^^5^ FAO also stimulates ketogenesis where the product, ketone bodies, can be passed to the photoreceptors as another source of energy.^3^^,^^6^^–^^8^ Perturbations to this system, such as RPE cells becoming more glycolytic, may result in retinal degeneration.^9^^–^^12^

Although FAO has a role in retinal function, it is not clear if it is required for overall vision. Interestingly, in most genetic disorders where FAO is disrupted, patients do not present with vision loss suggesting that impaired FAO alone does not cause retinal degeneration. The only FAO disorder where patients present with chorioretinopathy is long-chain 3-hydroxyacyl-CoA dehydrogenase deficiency (LCHADD; OMIM# 609016). It is hypothesized that the chorioretinopathy is caused by an accumulation of hydroxyacylcarnitines and hydroxy-fatty acids, unique intermediates seen only in LCHADD, that are potentially toxic to the RPE. Therefore, studying vision in FAO disorders, particularly LCHADD, will help improve our understanding of the impact of FAO on retinal health as well as identify other pathogenic mechanisms involved in retinal degeneration.

The pathogenic variant, c.G1528C in *HADHA* which encodes the alpha subunit of the mitochondrial trifunctional protein (TFP), causes LCHADD in human patients. TFP is a heterotetramer made up of two alpha- and two beta-subunits and is responsible for the last three steps of long-chain FAO: enoyl-CoA hydratase, LCHAD, and thiolase (Figs. 1A, 1B). The G1528C pathogenic variant occurs in the LCHAD active site and hinders only the LCHAD activity, whereas the stability and other enzymatic activities of TFP remain mostly intact.^13^^,^^14^ This causes the unique accumulation of long-chain hydroxyacylcarnitines, which serves as a biomarker for LCHADD.

**Figure 1.**
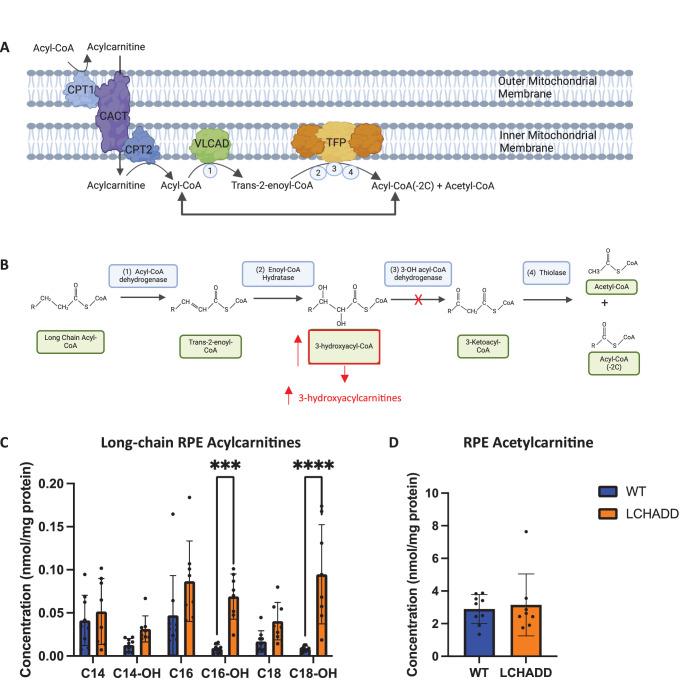
**LCHADD RPE displays an increase in hydroxyacylcarnitines but not a decrease in acetylcarnitine.** (**A**) Schematic of the proteins involved in long-chain fatty acid oxidation. (**B**) Enzymatic activity and intermediates involved in long-chain fatty acid oxidation. Red indicates the LCHADD G1528C pathogenic variant inhibits only the LCHAD activity and results in an accumulation of long-chain 3-hydroxyacylcarnitines, which serves as a biomarker for LCHADD. Figure was created using Biorender. (**C,** **D**) In isolated RPE/sclera samples, LCHADD (*n* = 8) compared to WT (*n* = 9) have (**C**) an increase in long-chain hydroxyacylcarnitines, suggesting a block in FAO at the LCHAD step. LCHADD samples have (**D**) equivalent acetylcarnitine, which is a marker of acetyl-CoA, and suggests that LCHADD RPE may not have an energy deficiency. Data presented as mean ± SD. A 1-way ANOVA with Sidak's multiple comparison post hoc test was used to calculate acylcarnitine statistics. An unpaired *t*-test was used to calculate acetylcarnitine statistics. * *P* < 0.05, **** *P* < 0.0001, ns = not significant.

The progression of chorioretinopathy in patients with LCHADD has been characterized. Young patients with LCHADD first present with pigment clumping and hypopigmentation at the posterior pole on fundus images, indicating that the loss of the RPE initiates the chorioretinopathy. Patients then develop atrophy of the choroid at the posterior pole and report poor night vision. Finally, patients lose photoreceptors, RPE, and choroid in the central fundus which progresses to blindness.^15^^,^^16^ The current treatment for patients with LCHADD is dietary management consisting of fasting avoidance and a diet low in long-chain fats supplemented with medium-chain triglycerides or triheptanoin (odd-chain medium length fatty acids) that provide a fatty acid substrate that can be used for energy. Although treatment can delay or slow progression, the diet does not prevent the chorioretinopathy, highlighting a need for a retina-specific treatment for LCHADD chorioretinopathy.^17^^–^^20^

Unfortunately, research into LCHADD chorioretinopathy has been limited due to the lack of model organisms. To address this problem, we recently reported a novel LCHADD mouse model that is homozygous for the c.G1528C variant in *Hadha* and recapitulated many of the human phenotypes. The LCHADD mouse model has decreased FAO, an accumulation of circulating hydroxyacylcarnitines, cardiomyopathy, and a reduced ability to exercise. Additionally, it displays retinal degeneration at 1 year of age.^21^ Here, we aim to further characterize the progression of the chorioretinopathy in the LCHADD mouse model by taking visual assessments at 3, 6, 10, and 12 months of age and aim to investigate the molecular mechanisms involved.

## Methods

### Animal Model

All animal procedures were reviewed and approved by the OHSU IACUC (eIACUC #B11243). Experiments were performed in accordance with AAALAC and ARRIVE guidelines. Experiments, except RNA-seq, was conducted using wildtype (WT) and LCHADD mice of mixed sex as no sex-specific differences were seen.

### Visual Performance using Optokinetic Tracking 

Optokinetic tracking (OKT) was performed as previously described with a camera calibrated to 22.08 pixels/cm.^21^ Right and left eyes were tested and recorded separately.

### Fundus Photography

Mice were anesthetized with an intraperitoneal (IP) injection of ketamine (90 mg/kg) and xylazine (9 mg/kg) followed by an injection of 20% dextrose (10 µL/kg). Eyes were dilated prior to imaging with 1% atropine, 1% tropicamide, and 2.5% phenylephrine, and then lubricated with 0.3% hypromellose eye gel. Fundus imaging was performed the same as previously described.^21^ After imaging, the mice were administered an IP injection of atipamezole (1 mg/kg) and 0.5% erythromycin ophthalmic ointment, and then they were placed on a heating pad.

### Spectral Domain Optical Coherence Tomography 

Mice were anesthetized and the eyes dilated the same as above. The spectral domain optical coherence tomography (SD-OCT) images were acquired with the SPECTRALIS HRA+OCT (Heidelberg Engineering; Heidelberg, Germany) using a 55 degrees lens. Fifteen linear horizontal B-scans generated from 1024 A-scans and averaging 50 ± 2 frames were captured across the IR retinal image. B-scans at approximately -1320, -220, 0, 220, and 1320 microns from the center of the optic nerve head were manually segmented to obtain the average thickness of the photoreceptors and RPE (Heidelberg Eye Explorer, version 1.10.12). Thickness was compared between LCHADD and WT retinas. Post-testing animal care was the same as stated above.

### Tissue Sectioning and Staining

Whole globes from 12 month old mouse eyes were enucleated, fixed in 4% paraformaldehyde (PFA), dehydrated in 30% sucrose, embedded in optimal cutting temperature compound, and snap-frozen. Embedded globes were sliced into 12-µm thick retinal cross sections (except for hematoxylin and eosin [H&E] staining) on a cryostat (Leica CM1860; Wetzlar, Germany) and stored at -20°C. All cryosections were thawed and rehydrated in 1× PBS prior to staining.

#### Hematoxylin and Eosin Staining

Retinal cryosections were stained with H&E and viewed on a Leica DMI3000B microscope (Leica Microsystems GmbH, Wetzlar, Germany) using the 40× objective. Chorioretinal phenotypes observed across each eye were characterized and frequencies of each phenotype was compared between WT and LCHADD samples.

#### Immunofluorescence on Retinal Cross Sections

For RPE65 staining, cryosections were permeabilized in 0.1% Triton X-100, blocked with 1% bovine serum albumin (BSA), and incubated in a recombinant rabbit anti-RPE65 primary antibody (1:100 dilution; Abcam, ab231782). For CD68 staining, cryosections were permeabilized and blocked in 1% BSA/0.3% Triton X-100, and incubated in a rabbit anti-CD68 (1:100 in 1% BSA/0.3% Triton X-100; Invitrogen, PA5-78996) primary antibody. All sections were incubated with a donkey anti-rabbit Alexa Fluor 647 (1:500 dilution; Invitrogen, A-31573) secondary antibody followed by DAPI.

Cross-sections were mounted using Fluoromount-G and imaged with a Leica Microsystems TCS SP8 X confocal system. Z-stacks were taken at 1-µm intervals. Post-imaging processing was conducted using ImageJ.

#### TUNEL Staining

Sections were stained with fluorescein-labeled dUTP according to manufacturer's protocol (Elabscience Bionovation; E-CK-A420). Slides were counter-stained with DAPI and mounted with Fluoromount-G. Imaging and post-imaging processing was conducted as described in the immunofluorescence (IF) section.

### Transmission Electron Microscopy

The 12 month old mice were anesthetized using isoflurane and perfused by flushing with PBS and administering fresh, EM-grade fixative (5% glutaraldehyde/2% paraformaldehyde in PBS; Electron Microscopy Sciences, cat #: 16220 and 15713). Mice were enucleated, the lens were removed, and samples were stored in fixative at 4°C.

Samples were processed in a Pelco Biowave Pro+ microwave with a SteadyTemp Pro chiller. The tissue was stained with an aqueous solution of 2% osmium tetroxide and 1.5% potassium ferricyanide, followed by staining with 1% uranyl acetate. The tissue was dehydrated in an increasing acetone series followed by infiltrated in EMbed812 resin. The tissue was flat embedded in coffin molds and polymerized in a 60°C oven. Once polymerized, the capsules were removed and sectioned. Sections were collected on slot grids and imaged on a Tecnai T12 transmission electron microscope equipped with an AMT Nanosprint12 camera, at 120 kV.

### Acylcarnitine Detection

The 15 month old mouse eyes were enucleated and RPE/sclera was isolated.^22^ The samples were flash frozen in liquid nitrogen and stored at -80°C. Samples were resuspended in sterile 1× PBS and homogenized using a pellet pestle motor and brief sonication. Acylcarnitines were analyzed by flow injection analysis tandem mass spectrometry (FIA-MS/MS) and quantified as previously published.^23^^,^^24^

### RNA Sequencing

RPE/scleras of 12 month old mice were isolated and stored like Acylcarnitine Detection samples. Samples were mechanically disrupted with the TissueLyser in QIAzol, run through a QIAshredder, and incubated in Proteinase K. RNA was isolated following RNeasy Micro (Qiagen, 74104) protocol with modifications added for fibrous tissue and an on-column DNase treatment using a QIAcube isolation robot. RNA was eluted in nuclease-free water.

Bulk RNA sequencing (RNA-seq) libraries were prepared using the TruSeq Stranded mRNA Library Prep Kit (Illumina). Briefly, RNA was profiled for integrity on a 2200 Bioanalyzer (Agilent). Poly(A)+ RNA was isolated using oligo-dT magnetic beads and then fragmented using divalent cations and heat. Fragmented RNA was converted to double stranded cDNA using random hexamer priming. The second strand was synthesized with dUTP in place of dTTP to prevent priming from this strand during amplification. The cDNA was ligated to adapters with dual unique indexes and amplified by limited rounds of polymerase chain reaction. The amplified material was cleaned using AMPure XP Beads (Beckman Coulter). The final library was profiled on a 4200 Tapestation (Illumina) and quantified using real time PCR with an NGS Library Quantification Kit (Roche/Kapa Biosystems) on a StepOnePlus Real Time PCR Workstation (Thermo/ABI).

Libraries were sequenced on a NovaSeq 6000 (Illumina) running RTA version 3.4.4. BCL files were demultiplexed using bcl2fastq version 2.20.0.422 (Illumina). Sequencing quality was checked with FastQC (Babraham Bioinformatics). Libraries were trimmed using Trimmomatic 0.36 (Bolger et al. 2014, Bioinformatics 30, 2114-2120), then aligned using STAR 2.5.0a (Dobin et al. 2013, Bioinformatics 29, 15-21) and reference genome GRCm38. SAMtools 1.5 (Li et al. 2009, Bioinformatics 25, 2078-2079) was used to generate the bam formatted alignment.

RNA-seq was analyzed using the Galaxy Project.^25^ “Reference-based RNA-Seq data analysis” and “Visualization of RNA-Seq results with Volcano Plot” tutorial was followed.^26^^–^^29^

### Reverse Transcription-Quantitative Polymerase Chain Reaction 

RNA extracted for the RNA-seq was also used to make cDNA using the High Capacity RNA-to-cDNA Kit (Thermofisher). The quantitative PCR (qPCR) was performed on the QuantSudio 5 Real-Time PCR System (Thermofisher) with approximately 10 ng of cDNA, 250 nm of forward/reverse primers, and Power Syber Green PCR Master Mix (Thermofisher). Amplification was performed at 95°C for 10 minutes, followed by 40 cycles of 95°C for 15 seconds, 60°C for 15 seconds, and 60°C for 45 seconds. Primer sequences were:

*B-actin* Forward: 5′-CCCGGGCTGTATTCCCCTCCAT-3′
*B-actin* Reverse: 5′-TGGGCCTCGTCACCCACATAGG-3′
*Mitf* Forward: 5′-TGAGGAGCAGAGCAGGGCAGAG-3′
*Mitf* Reverse: 5′-CAAGGCCGGATCCATCAAGCCC-3′

### Mitochondrial Analysis

The size of the mitochondria was calculated by tracing mitochondria on transmission electron microscopy (TEM) images (6800× magnification) and measuring the area using FIJI. Four TEM images per eye were counted, the total number of mitochondria counted per animal ranged from 54 to 130, and the total number of mitochondria counted for WT and LCHADD animals were 276 and 266 mitochondria.

The mitochondria copy number was determined by isolating DNA from RPE/sclera using the DNeasy Blood and Tissue Kit (Qiagen, 69504). Quantitative PCR was performed using DNA, forward/reverse *Mt-Nd6* and *Polb* primers,^30^ and Power SYBR Green PCR Mastermix (ThermoFisher Scientific, 4368708) on a QuantStudio 5 Real-Time PCR System (Thermofisher). Amplification was performed at 95°C for 10 minutes, followed by 40 cycles of 95°C for 15 seconds, 53°C for 15 seconds, and 60°C for 45 seconds. The relative mitochondrial copy number was calculated as previously published.^31^

### Statistical Analysis

Statistical analysis was performed using GraphPad Prism 10.2 software. All data are presented as mean ± SD. Different statistical tests were used. A 1-way ANOVA was used for long-chain acylcarnitine analysis; a unpaired 2-tail *t*-test was used for acetylcarnitine, macrophage, TUNEL, and mitochondrial copy number analyses; a mixed-effects model with Sidak's multiple comparison post hoc test was used for visual performance analysis; a 2-way ANOVA with Sidak's multiple comparison analysis was used for retinal thickness analysis; a 1-tailed *t*-test was used for *Mitf* RT-qPCR analysis; and a nested *t*-test was used for mitochondrial size analysis. Figures also indicate when each test was used. The *P* values of less than 0.05 were considered statistically significant.

## Results

To test if LCHADD RPE has reduced LCHAD activity and an impaired FAO, we measured acylcarnitine concentrations in RPE/sclera samples isolated from 15 month old WT and LCHADD mice of mixed sexes. LCHADD RPE/sclera show a statistically significant increase in long-chain hydroxyacylcarnitines compared to WT RPE/sclera (Fig. 1C). Specifically, there was a 7-fold and a 5-fold increase in C16-OH and C18-OH. Acetylcarnitine is not different between LCHADD and WT RPE/sclera (Fig. 1D). Acetylcarnitine is a marker for acetyl-CoA, the product of FAO and glycolysis that is important for the citric acid cycle and subsequently ATP production. Although the high hydroxyacylcarnitines suggest LCHADD RPE/sclera have a block at the LCHAD step, the equivalent acetylcarnitine levels indicate there may not be an energy deficiency.

**Figure 2.**
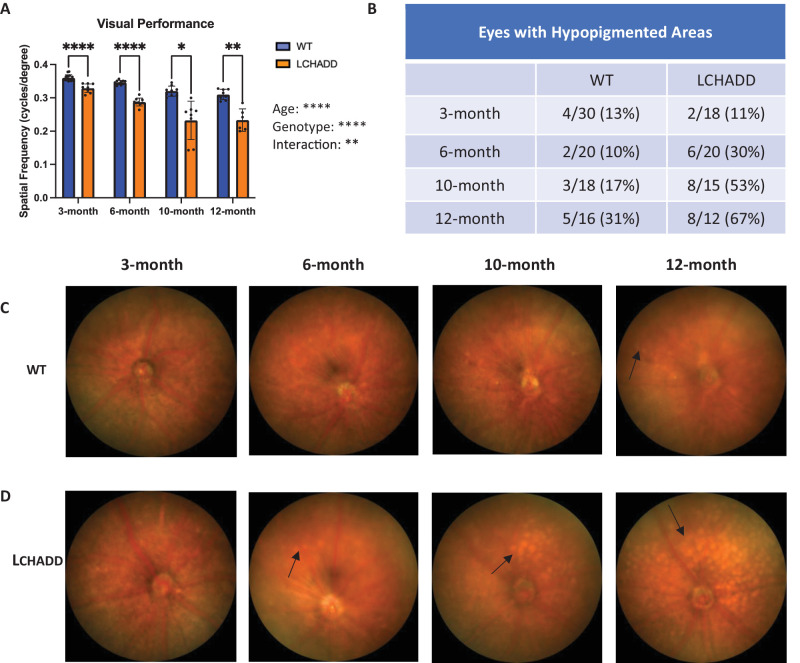
**LCHADD mice display a decreased visual performance due to increased RPE degeneration, starting at 6** **months of age, that progressively worsens over time.** (**A**) OKT measurements in LCHADD mice (*n* = 6–10 mice) show a significantly lower visual performance compared to WT mice (*n* = 8–12 mice) that progressively decreases overtime. Data presented as mean ± SD. Statistics were calculated by a mixed–effects model and Sidak's multiple comparisons post hoc test. (**B**) Percentage of fundus images with white, hypopigmented spots in WT and LCHADD mice at 3, 6, 10, and 12 months of age. (**C**) Examples of WT eyes at 3, 6, 10, and 12 months of age show white, hypopigmented spots are minimal in WT eyes even at 12 months of age. (**D**) Examples of LCHADD eyes at 3, 6, 10, and 12 months of age that demonstrate white, hypopigmented spots that first appear at 6 months of age and increase over time, suggesting a progressive increase in RPE degeneration. Black arrows = hypopigmented spots. * *P* < 0.05, ** *P* < 0.01, **** *P* < 0.0001.

We have previously reported that LCHADD mice have a decrease in visual performance at 12 months of age.^21^ To characterize the changes in visual performance over time, we measured the spatial frequency of WT and LCHADD mice at 3, 6, 10, and 12 months using OKT (Fig. 2A). The spatial frequency in LCHADD mice, whereas statistically significant, is only slightly decreased when compared to WT (0.33 vs. 0.36 cycles/degree) at 3 months. This difference increased to 0.06 cycles/degree at 6 months (0.29 vs. 0.35 cycles/degree), which is statistically significant and more biologically relevant than at 3 months. The difference between LCHADD and WT visual performance was the largest at 10 months (0.23 vs. 0.32 cycles/degree) and remained the same at 12 months. This suggests that, by 6 months, LCHADD mice have decreased visual performance that progressively worsens compared to WT mice.

**Figure 3.**
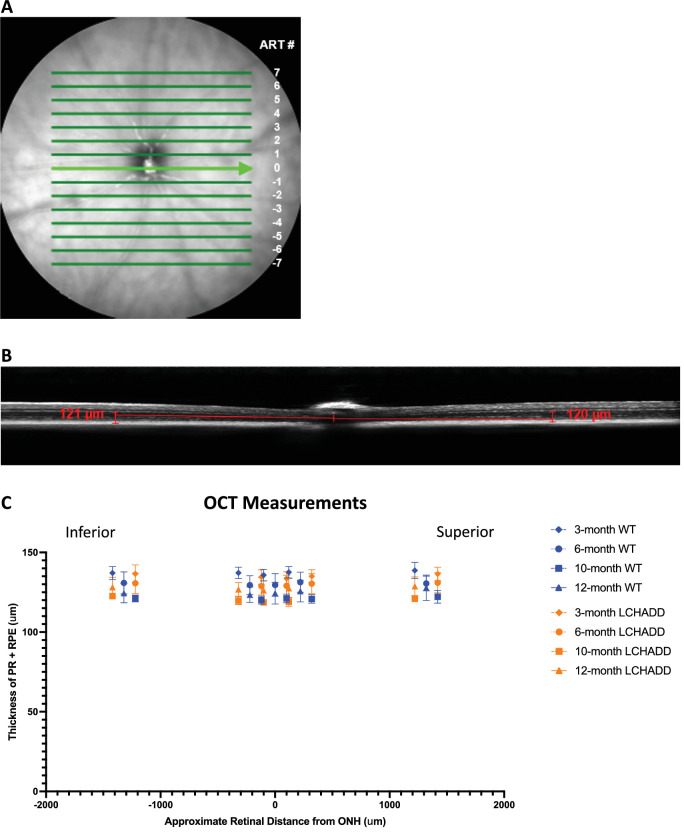
**No difference in retinal thickness between WT and LCHADD mice at any age.** (**A**) A representative IR fundus image showing OCT image acquisition. The *green lines* demarcate the B-scans acquired and each are approximately 220 µm apart. The ART # is used as a label for each location. ART #0 scans through the center of the optic nerve head. (**B**) A representative 3 month LCHADD OCT image. The photoreceptor (PR) and RPE thickness was measured approximately 520 µm on the temporal and nasal side. The measurements were averaged together. (**C**) The thickness of the PR and RPE layers in WT (*n* = 7–11 mice) and LCHADD mice (*n* = 3–8 mice) at 3, 6, 10, and 12 months of age. The thickness of PR and RPE were measured at ART #s -6, -1, 0, 1, and 6 (approximately -1320, -220, 0, 220, and 1320 µm from the center of the optic nerve head). Symbols were slightly displaced along the x-axis to allow for better visualization. There was no difference in PR and RPE thickness between WT and LCHADD mice at any age. Data presented as mean ± SD. A 2-way ANOVA with a Sidak's multiple comparison analysis was used to calculate statistics for each age.

Next, fundus imaging was performed in WT (Fig. 2C) and LCHADD (Fig. 2D) mice at 3, 6, 10, and 12 months of age. RPE degeneration was evident and characterized by hypopigmented areas. Starting at 6 months of age, the percentage of LCHADD eyes with hypopigmentation was at least twice the number of WT eyes. In addition, the percentage of LCHADD eyes with hypopigmentation continued to increase from 11% at 3 months to 67% at 12 months, whereas the percentage of WT eyes with pigmentation remained between 10% and 17% until 10 months and only increased to 30% at 12 months (Fig. 2B). This suggests LCHADD mice have increased RPE degeneration compared to WT mice starting at 6 months that worsens over time. Optical coherence tomography (OCT) was used to look at overall retinal degeneration. Like previously reported findings, there was no difference in the thickness of the photoreceptor and RPE layers between WT and LCHADD mice (Fig. 3), indicating photoreceptors have not degenerated by 12 months.^21^ TUNEL staining on 12-month LCHADD central retinal cross-sections do indicate increased TUNEL-positive cells in the cross-sections compared to WT; however, this is not statistically significant (*P* = 0.06) and the total number of TUNEL-positive cells is low (Supplementary Fig. S1). This indicates that LCHADD mice may have minor degeneration in other layers of the retina compared to WT but not enough to alter the retinal structure. Overall, LCHADD mice have progressive vision loss and RPE degeneration compared to WT starting at 6 months of age, although not an overall change in retinal thickness.

**Figure 4.**
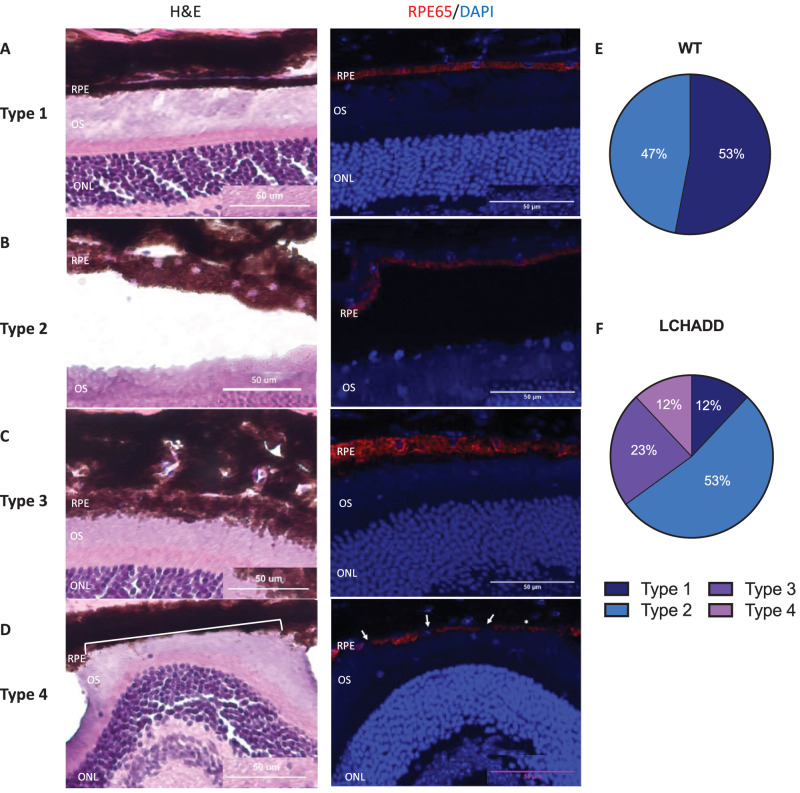
**Twelve** **month LCHADD RPE show increased RPE disruptions ranging from RPE vacuoles to RPE loss in areas.** Four different types of RPE were seen from 12 month WT (*n* = 15 central retina cross section from 5 eyes) and LCHADD (*n* = 17 central retina cross section from 6 eyes) retinal cross sections stained with H&E or RPE65/DAPI (*red/blue* fluorescence). (**A**) Type 1 RPE were defined as normal, healthy RPE. (**B**) Type 2 RPE were defined as RPE with vacuoles in the RPE on H&E staining, whereas RPE65 showed a normal RPE. (**C**) Type 3 RPE were defined as RPE that had a loss of rigidity and disintegration RPE on both H&E and RPE65 staining. (**D**) Type 4 RPE were defined as areas where there was a loss of RPE on H&E staining (*white bracket*) and either significant thinning (*white asterisk*) or complete loss (*white arrow*) of RPE65 staining. (**E,** **F**) When the types of RPE were quantified, LCHADD samples had a higher percentage of RPE that were disrupted and had more severe disruptions (types 3 and 4) when compared to WT mice.

To further analyze the RPE layer, H&E staining and RPE65 IF showed that, at 12 months of age, LCHADD mice had increased RPE disruptions and degenerations compared to WT mice. The RPE was characterized into four types: type 1 indicates normal RPE where H&E and RPE65 staining show a consistent, continuous RPE layer (Fig. 4A); type 2 indicates RPE that developed vacuoles seen in H&E staining (Fig. 4B); type 3 indicates RPE with a loss of boundary rigidity and lack of uniform pigmentation in both stains (Fig. 4C); and type 4 indicates RPE loss as seen from a loss of pigmentation on H&E or a severe thinning/complete absence of RPE65 fluorescence (Fig. 4D). When quantified, WT mice had minimal RPE disruption where 53% of the samples had normal RPE and 47% of the RPE had vacuoles (types 1 and 2; Fig. 4E). In contrast, 88% of LCHADD samples had some degree of RPE disruption and 35% of samples had a loss of boundary rigidity and/or RPE loss (types 3 and 4; Fig. 4F). Interestingly, we saw a large number of choroid vacuoles in the LCHADD samples compared to WT samples (44% vs. 20%; Supplementary Fig. S2). Further analysis is needed to determine the etiology and significance of these vacuoles.

**Figure 5.**
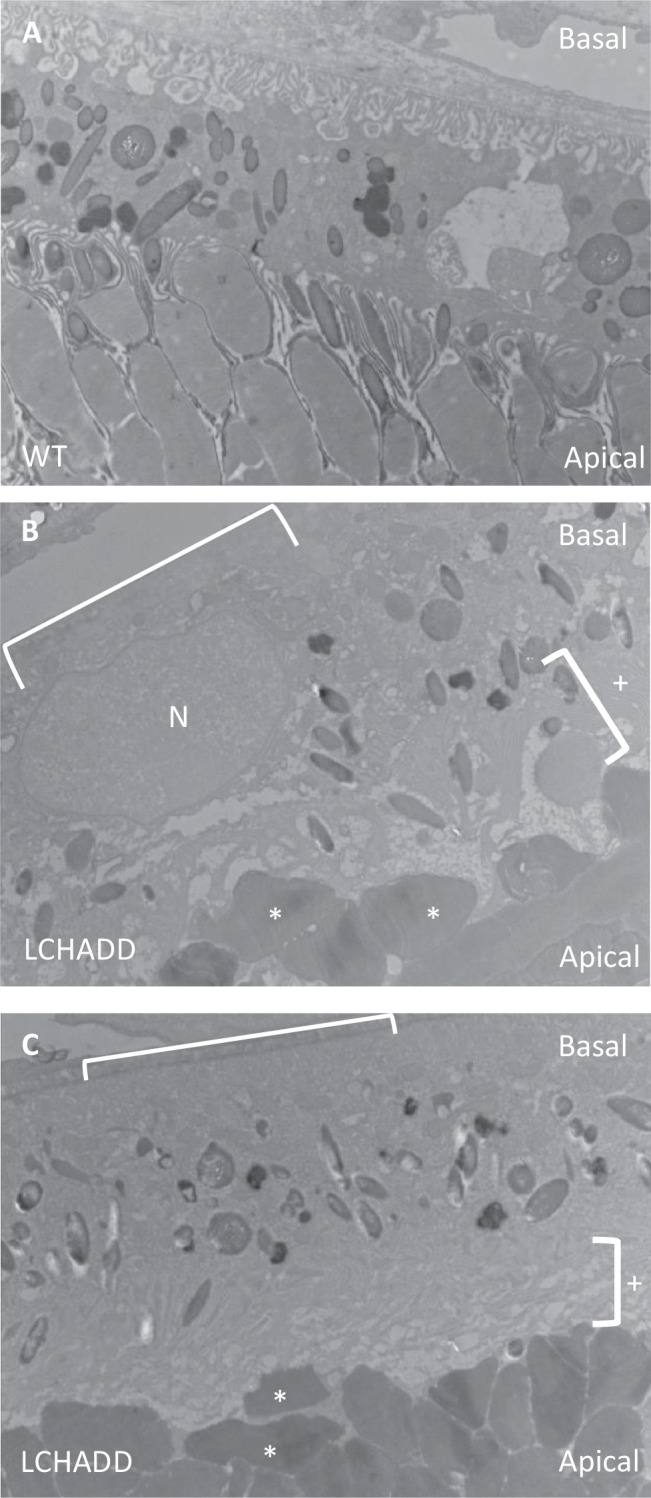
**TEM imaging shows 12** **month LCHADD RPE display an altered RPE structure.** (**A**) RPE from 12 month old WT mice have a relatively normal RPE structure with basal infoldings and apical microvilli. (**B,** **C**) Representative images of LCHADD RPE, on the other hand, display a loss of basal infoldings (*bracket only*), loss of apical microvilli (*bracket with plus sign*), and abnormal photoreceptor outer segments that are horizontal to the RPE (*white asterisk*). N = nucleus and V = vacuole.

Transmission electron microscopy (TEM) further suggests a change in RPE structure of 12 month old LCHADD mice. WT RPE have a normal structure with some vacuoles present (Fig. 5A, Supplementary Figs. S3A-D). LCHADD RPE appear to have a loss of basal infoldings, loss of apical microvilli intertwining with photoreceptor outer segments, and photoreceptor outer segments that are abnormal and horizontal to the RPE (Figs. 5B, 5C, Supplementary Figs. S3E–H). TEM imaging also suggests mitochondria in LCHADD RPE trend larger than WT; however, this is not statistically significant (*P* = 0.11; Supplementary Figs. S4A–C). There was no difference in the number of mitochondria between WT and LCHADD RPE/sclera (Supplementary Fig. S4D). Future experiments, such as TEM imaging of RPE with higher magnification, are needed to further explore the mitochondrial morphology and function in LCHADD RPE. Overall, TEM indicates an altered RPE structure confirming H&E and RPE65 staining.

**Figure 6.**
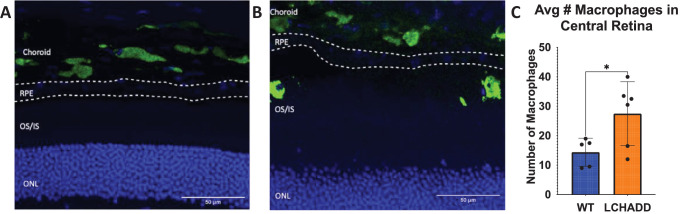
**Twelve** **month** **old** **LCHADD mice have increased infiltration of macrophages into the subretinal space when compared to WT mice suggesting increased inflammation and cell death.** (**A,** **B**) Representative images of (**A**) WT and (**B**) LCHADD retinal cross sections stained with CD68 (*green*) and DAPI (*blue*) suggest an increased number of macrophages that infiltrate the subretinal space of LCHADD mice. (**C**) Quantification of the average number of macrophages in the subretinal or neural retinal space in the central retina. This was calculated by averaging the number of macrophages taken from two central retinal cross sections taken from 12 month old LCHADD (*n* = 6) and WT (*n* = 5) mice. Data presented as mean ± SD. Statistics were measured using a two-tail *t*-test. * *P* < 0.05.

Increased macrophages in the RPE and subretinal space of a deceased 14 month old patient with LCHADD presenting with early-stage chorioretinopathy was previously reported.^32^ An increase in CD68+ macrophages in the subretinal space have also been associated with ectopic RPE and RPE dystrophy in animal models of retinal degenerations, suggesting a role of CD68+ macrophages in the retinal inflammation commonly seen in retinopathies.^33^ To determine if similar macrophage infiltration exists in the LCHADD mouse model, we stained retinal cross sections for CD68. The average number of macrophages present in the central retina of WT and LCHADD mice were 14 vs. 27, suggesting increased macrophages in LCHADD mice (Figs. 6A–C).

**Figure 7.**
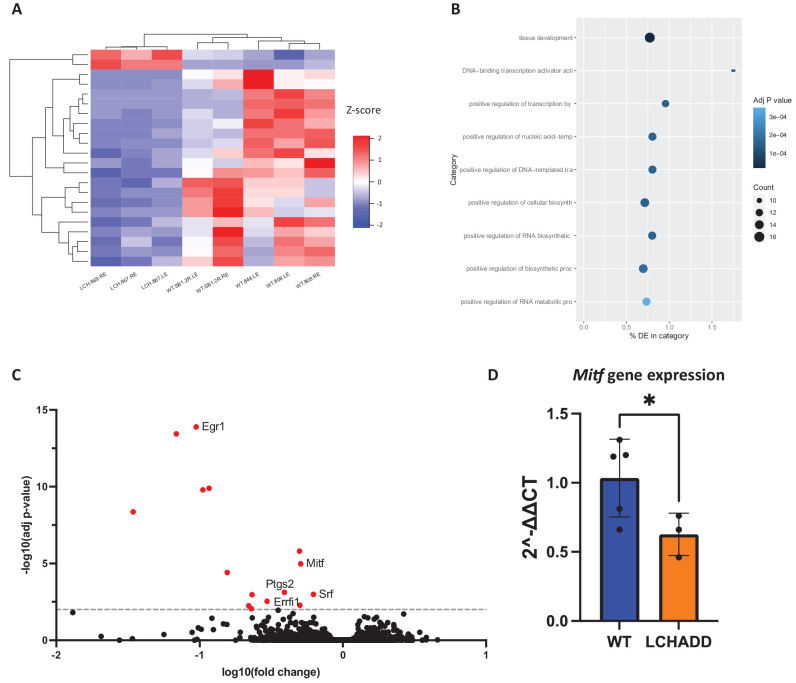
**RNA-sequencing of 12** **month** **old** **male WT (*n* = 5) and LCHADD (*n* = 3) RPE/sclera highlight genes that are differentially expressed in LCHADD RPE/sclera samples.** (**A**) Heatmap of Z-scores calculated from RNA-sequencing of 12 month old male WT and LCHADD mice. (**B**) Top 10 gene ontology (GO) terms based on the DEG in LCHADD mice. The most over-represented category is tissue development. (**C**) Volcano plot of differentially expressed genes (DEGs) found in LCHADD mice compared to WT mice. *Red*, highlighted points are significant DEGs with a -log10 (adjusted [adj] *P* value) > 2. Points with labels indicate genes that have been previously studied in the retina. These genes have been shown to be involved in vasculature and angiogenesis (*Egr1*, *Errfi1*, *Srf*, and *Ptgs2*), and RPE function (*Mitf*). (**D**) RT-qPCR confirms that *Mitf* gene expression is decreased in 12 month old LCHADD RPE/sclera. Statistics were calculated using a one-tailed *t*-test. * *P* < 0.05.

Finally, RNA-sequencing on 12 month old male WT mice and LCHADD RPE/sclera were used to investigate molecular mechanisms involved in LCHADD chorioretinopathy. LCHADD RPE/sclera have differentially expressed genes (DEGs) compared to WT mice (Fig. 7A). There were 16 statistically significant DEGS, all of which were downregulated in LCHADD RPE/sclera, and the most common gene ontology term taken from the DEGs was tissue development (Figs. 7B, 7C, Supplementary Table S1). Interestingly, many of the significant DEGs have been previously studied in the context of retinal degeneration, specifically playing a role in angiogenesis (see Fig. 7C).^34^^–^^38^ One gene of particular interest, *Mitf*, is expressed solely in the RPE and plays a crucial role in RPE function.^39^^–^^42^ Loss of this gene has been connected to the loss of RPE pigmentation and the loss of RPE phenotype, thus supporting the results seen from H&E staining and TEM.^43^^,^^44^ RT-qPCR also showed *Mitf* gene expression in 12 month p;d LCHADD RPE/sclera was 60% lower compared to WT, confirming RNA-sequencing results (Fig. 7D). Overall, these data support that 12 month old LCHADD male mice have an altered gene expression in the RPE/sclera, and, in addition to RPE dysfunction, changes in the vasculature could be significantly contributing to the LCHADD chorioretinopathy.

## Discussion

LCHADD is a rare, long-chain FAO disorder that was first reported in 1989 and affects between 1:250,000 and 1:750,000 people depending on location.^45^^–^^47^ With more patients with LCHADD living past infancy due to early detection and better treatments, patients must cope with chronic, later-onset symptoms, such as chorioretinopathy, which are not well researched nor preventable. Studying the molecular mechanisms involved in LCHADD chorioretinopathy will not only allow for the development of a better treatment, but it will elucidate the role of FAO in the RPE, a current area of research, and identify other pathogenic mechanisms that result in retinal degeneration. Results presented here show that the recently reported LCHADD mouse model recapitulates early-stage LCHADD chorioretinopathy in patients and provides a model to study the molecular mechanisms involved in LCHADD chorioretinopathy.

The causes of LCHADD chorioretinopathy are currently unknown; however, many believe that an accumulation of toxic intermediates and/or an energy deficiency contribute to RPE degeneration. Recent research suggested that fatty acids are a major energy source in RPE and the loss of FAO in RPE cells disrupts the energy production in RPE. This negatively impacts the metabolic homeostasis in the retina, eventually leading to retinal degeneration.^1^^–^^12^ This hypothesis is challenged by the observation that LCHADD is the only FAO disorder that presents with chorioretinopathy. If an impaired FAO is sufficient in causing retinal degeneration, then one would expect other FAO disorders to also present with retinopathy. Because LCHADD is also the only FAO disorder that accumulates long-chain hydroxyacylcarnitines, it is hypothesized that hydroxyacylcarnitines are toxic to RPE cells. Previous reports have negatively correlated the sum of hydroxyacylcarnitines in plasma of patients with LCHADD with visual acuity.^17^ In our results, we see no change in acetylcarnitine levels but a significant accumulation of hydroxyacylcarnitines in RPE/sclera from 15 month old LCHADD mice. Acetylcarnitine is a marker for acetyl-CoA. Because acetyl-CoA is an important metabolic intermediate that regulates many cellular processes and signal transduction, the levels may reflect the metabolic state of the cells. As we do not see a decrease in acetylcarnitine levels, our data suggests that energy deficiency may not play a major role in LCHADD chorioretinopathy. In contrast, the higher level of hydroxyacylcarnitines is associated with vision loss indicating they are potentially toxic to the RPE. It is important to note that many different metabolic processes create and utilize acetyl-CoA and subcellular compartmentalization of acetyl-CoA can be a better gauge of cellular state^48^; therefore, more experiments need to be performed to confirm our findings including direct measurements of energy state, such as ATP, Krebs cycle intermediates, and lactate. In addition, whereas we expect hydroxyacylcarnitine accumulation in young mice because it can be detected in newborn patients with LCHADD, acylcarnitine concentrations will need to be measured in RPE/sclera from 3 to 6 month old mice to determine how they change overtime.

We show chorioretinopathy in the LCHADD mouse is initiated by RPE degeneration and there is increased macrophage presence in the subretinal space. Both findings are similar to what has been observed from patient studies^15^^,^^32^; however, this mouse model provides detailed information on the structural changes in LCHADD RPE. Our results suggest LCHADD RPE cells may be losing polarity and barrier function in LCHADD chorioretinopathy. RPE polarity is crucial in allowing the RPE to regulate transport, whether that be through trafficking proteins to apical or basal membrane, melanosome movement, or phagocytosing photoreceptor outer segments.^49^ The apical microvilli and basal infoldings play a role in regulating transport with the choroid and photoreceptor outer segments and many important proteins are localized to the apical or basal membranes.^50^ The loss of the basal infoldings and apical microvilli suggest that transport and integrity of the cell boundaries are compromised (see Fig. 5). This compromised transport and loss of barrier function can result in abnormal uptake, such as the increased presence of vacuoles seen in type 2 RPE, and loss of boundaries and structure of RPE cells, as seen from the loss of rigidity in type 3 RPE. The altered structure can result in RPE dysfunction and, eventually, degeneration. The downregulation of *Mitf* further supports RPE dysfunction and loss of boundary formation as this gene has been shown to be crucial in RPE development and differentiation and transepithelial transport.^41^^,^^42^ Overall, the LCHADD mouse chorioretinopathy mirrors what has been observed in patients with LCHADD and provides a useful model to study the molecular mechanisms involved with the chorioretinopathy.

Unexpectedly, we do see some retinal degeneration in the WT mice at 12 months of age. One possible explanation could be natural degeneration in C57BL/6J mice, which is the background of the LCHADD mice.^51^ Another potential explanation could be a result of phototoxicity.^52^ While the mice were housed in conditions where they were exposed to less than 6 lux of light, which is known to not cause phototoxicity, they could have been exposed to high light intensities during transportation or testing that caused damage. Finally, although none have been identified yet, this mouse line could be more susceptible to retinal degeneration because of off-target mutations introduced by CRISPR/Cas9, which was used to create this LCHADD line.^21^ This is an important factor to consider for future experiments on older mice.

This study has a few limitations. First, the progression of chorioretinopathy in the LCHADD mouse is slower and less severe when compared to patients. Patients with LCHADD present with chorioretinopathy usually in the first decade and quickly progress to the loss of the photoreceptors. This is evident as retinal thinning seen using OCT.^53^ In this mouse model, we did not see a significant change in the photoreceptor layer nor any discernable changes in OCT, indicating that even at 12 months of age the LCHADD mice have early-stage chorioretinopathy (approximately stage 2A).^16^ One potential explanation for this is that the mice are not stressed. During their 12 months, these mice had no metabolic crises initiated from exercising/fasting/illness, which are commonly seen in patients. Maintaining a low-fat diet (approximately 10% of caloric intake) and avoiding metabolic crises have been shown to delay or slow the progression of the chorioretinopathy in patients, suggesting that these moments of stress accelerate the progression potentially by significantly increasing hydroxyacylcarnitine levels.^17^^–^^19^ As these mice were never stressed (fasted/exercised/sick) and they normally eat a low-fat diet (approximately 16% of calories provided by fat; Formulab Diet 5008, Irradiated), it is possible they are not being sufficiently challenged and we could potentially accelerate the progression by stressing the mice. Another limitation is that patients with LCHADD can develop choroid neovascularization (CNV).^54^^,^^55^ Whereas we report here that LCHADD mice have increased vacuoles in the choroid and many DEGs in LCHADD mice have been reported to play a role in angiogenesis and vasculature, the quality of the choroid was outside of the scope of this paper.^34^^–^^38^^,^^42^ Therefore, it will be important to use appropriate techniques, such as OCT angiography, to assess the vasculature of this LCHADD mouse.

Because the current dietary treatment does not prevent LCHADD chorioretinopathy, this LCHADD mouse model will be crucial in the development of new retinal-specific treatments. Gene therapy has been investigated as a potential treatment for FAO disorders and is a promising treatment for LCHADD chorioretinopathy.^56^^–^^59^ Because LCHAD is an autosomal recessive, monogenic disorder that is caused by a loss of LCHAD activity, adding a functional *HADHA* gene to the RPE may be sufficient in preventing the chorioretinopathy by restoring LCHAD activity and FAO in the retina and, ultimately, decreasing hydroxyacylcarnitines. This model will allow us to evaluate gene therapy as a potential treatment for LCHADD chorioretinopathy.

## Conclusions

Overall, we report that the new LCHADD mouse model develops early-stage chorioretinopathy and is a promising model when studying LCHADD chorioretinopathy. Similar to humans, chorioretinopathy in LCHADD mice is likely initiated by a loss of the RPE layer. This study provides evidence of the RPE losing barrier function and polarity, as seen on TEM and RNA-seq. Although there is some optimization required with this model, such as increasing the rate of progression, this model will advance chorioretinopathy research and ultimately test the efficacy of therapies that can preserve visual function for patients living with LCHADD.

## Supplementary Material

Supplement 1
