# Supplementary material for: The LCHADD Mouse Model Recapitulates Early-Stage Chorioretinopathy in LCHADD Patients
Source: Invest Ophthalmol Vis Sci. 2024 Jun 21;65(6):33. doi: 10.1167/iovs.65.6.33 (PMC11193142; doi:10.1167/iovs.65.6.33)
Supplement: Supplement 1 [file iovs-65-6-33_s001.pdf]

Supplemental Figures:

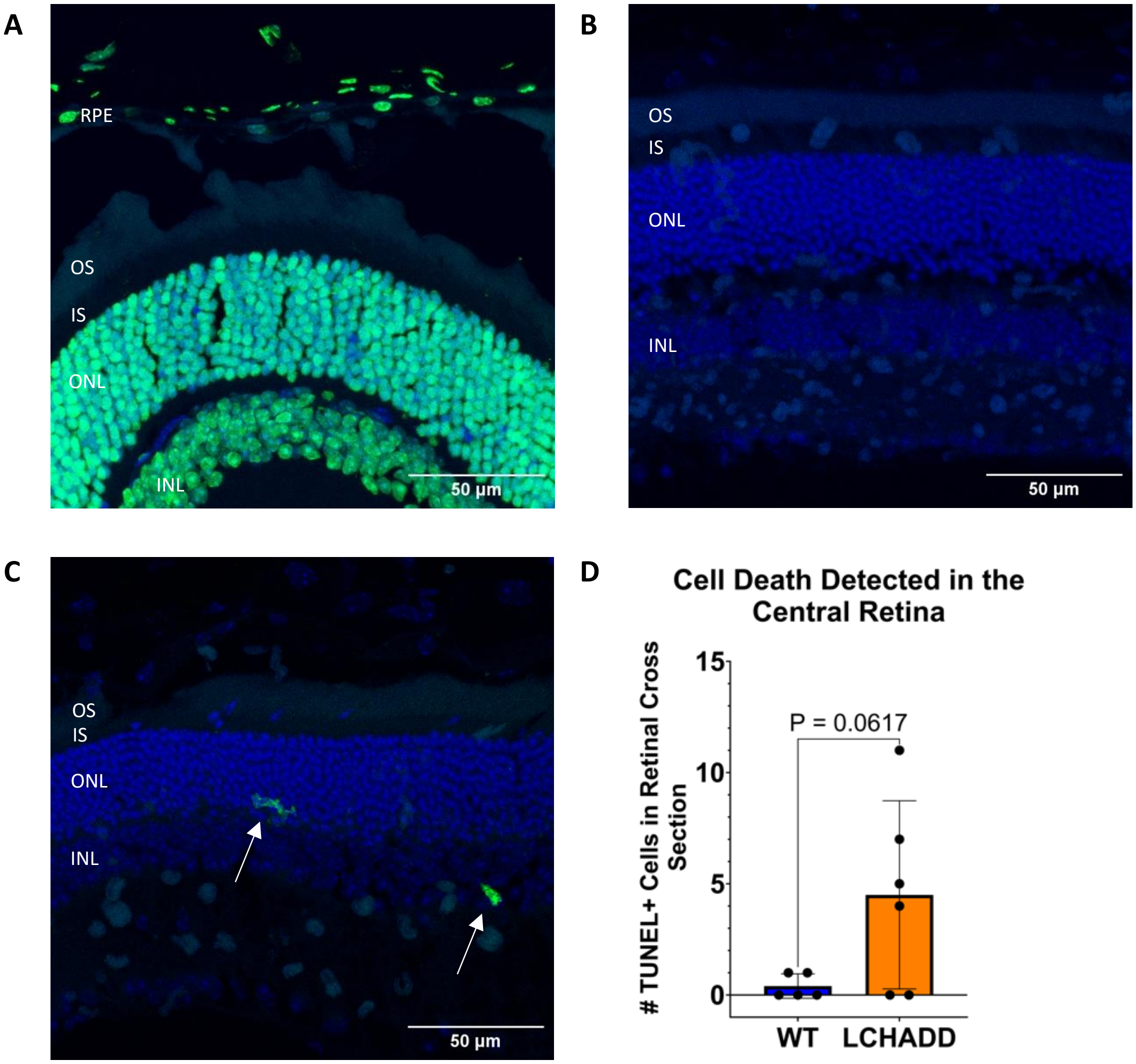

Supplemental Figure 1: There is no statistically significant difference in apoptotic cells between the central retina cross-sections of 12-month WT and LCHADD mice, indicating no significant retinal degeneration. (A-C) Representative images of (A) positive control, (B) 12-month WT, and (C) 12-month LCHADD retinal cross-sections stained with TUNEL staining (green) and DAPI (blue). Both WT and LCHADD retinal cross-sections have few apoptotic cells (white arrows), suggesting no retinal degeneration. (D) Quantification of the number of TUNEL-positive cells in the retinal cross-sections from 12-month WT and LCHADD central retina cross-sections. LCHADD mice have increased TUNEL-positive cells compared to WT cross-sections. Data presented as mean ± SD. Stats were measured using a two-tail T-test.

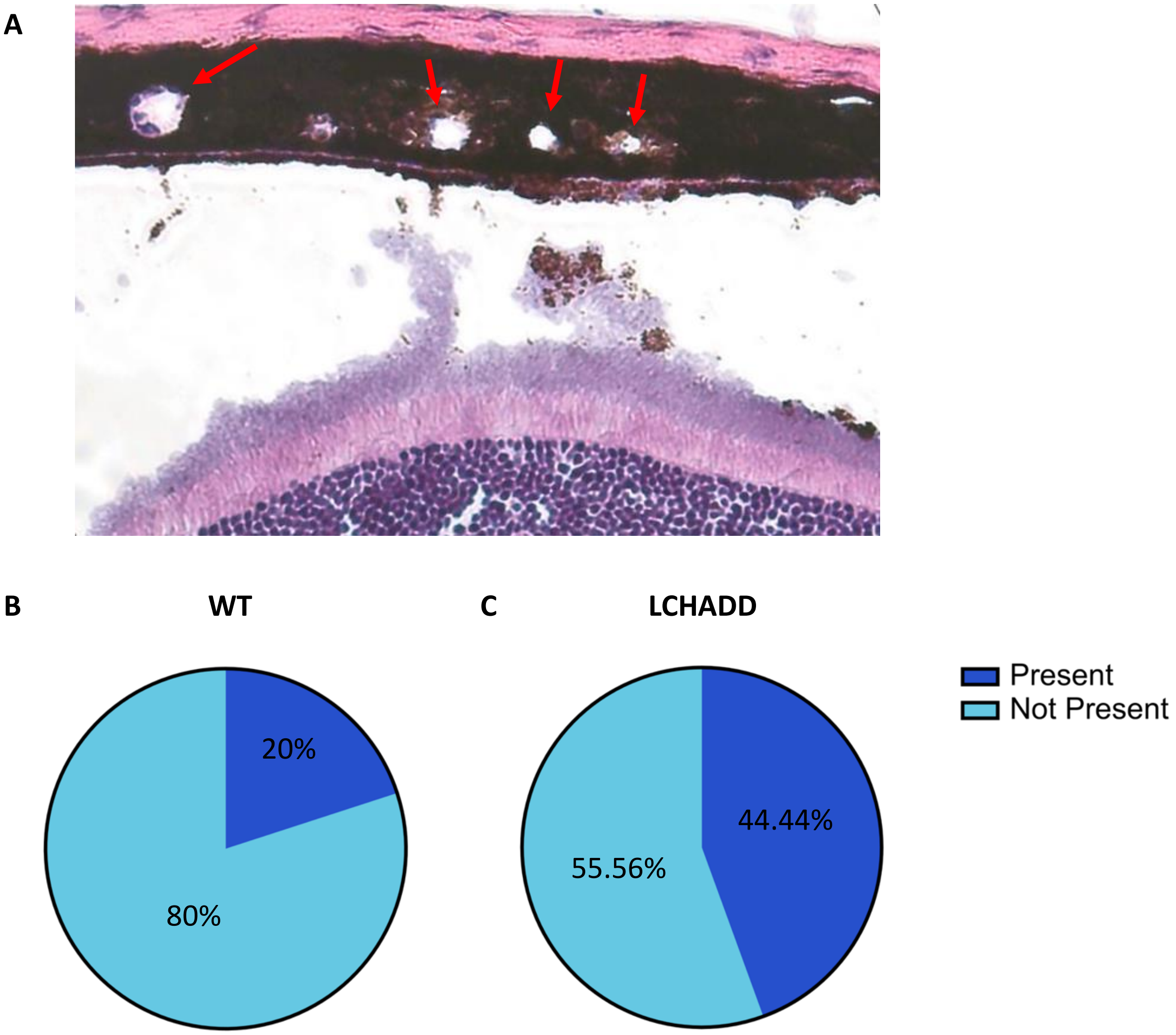

Supplemental Figure 2: 12-month LCHADD mice have increased vacuoles in the choroid compared to WT mice. (A) An example of vacuoles (red arrows) seen in choroid of 12-month LCHADD mice. (B-C) Percentage of H&E-stained retinal cross sections with vacuoles in choroid in 12-month (B) WT and (C) LCHADD mice.

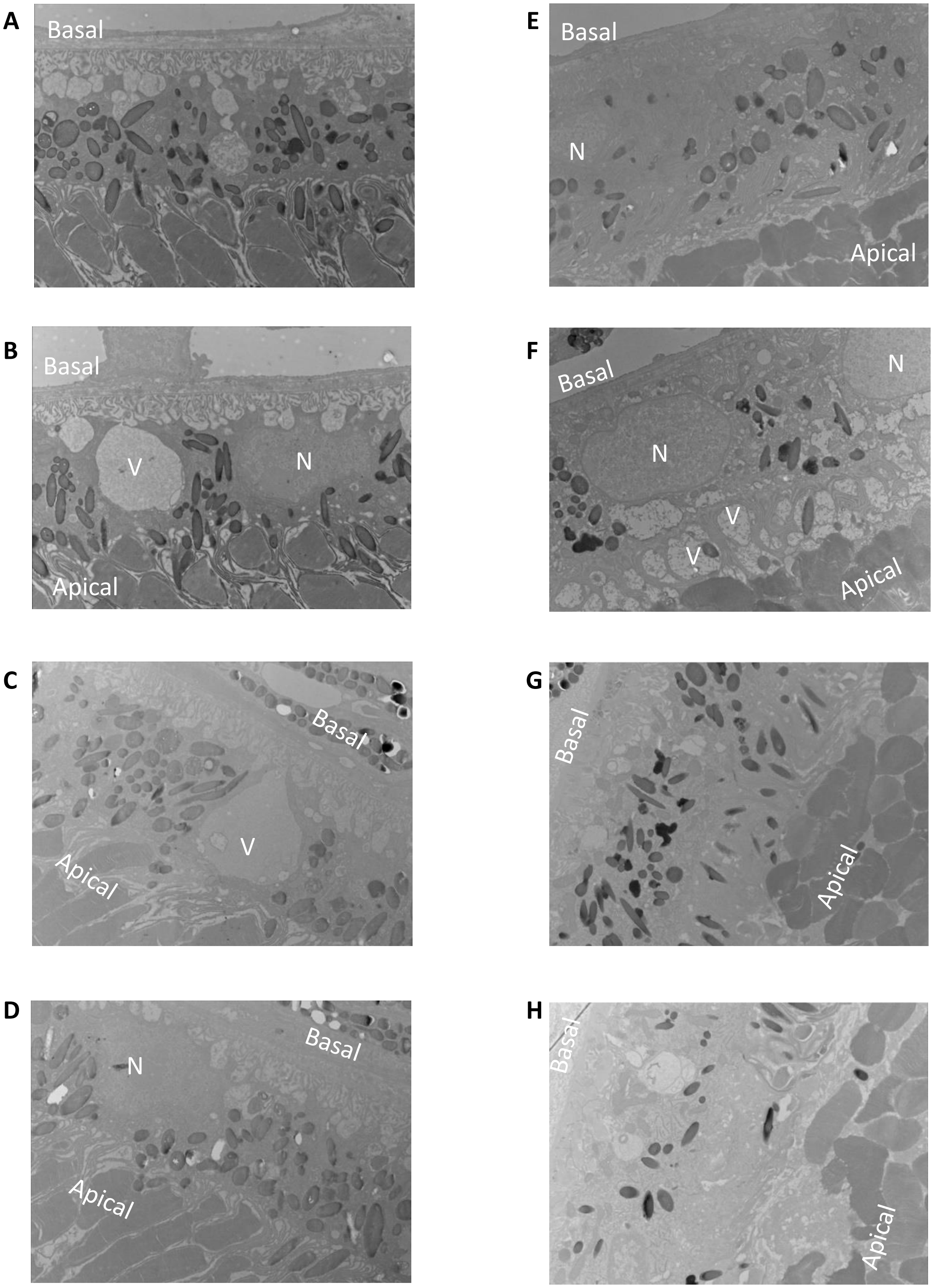

Supplemental Figure 3: Additional TEM images of 12-month WT and LCHADD RPE. (A-D) Additional WT RPE (n=2) that show a normal RPE that has basal infoldings and apical microvilli. (E-H) Additional LCHADD RPE (n=2) that have a loss of basal infoldings, loss of apical microvilli, and abnormal photoreceptor outer segment that are horizontal to RPE. N= nucleus and V= vacuoles.

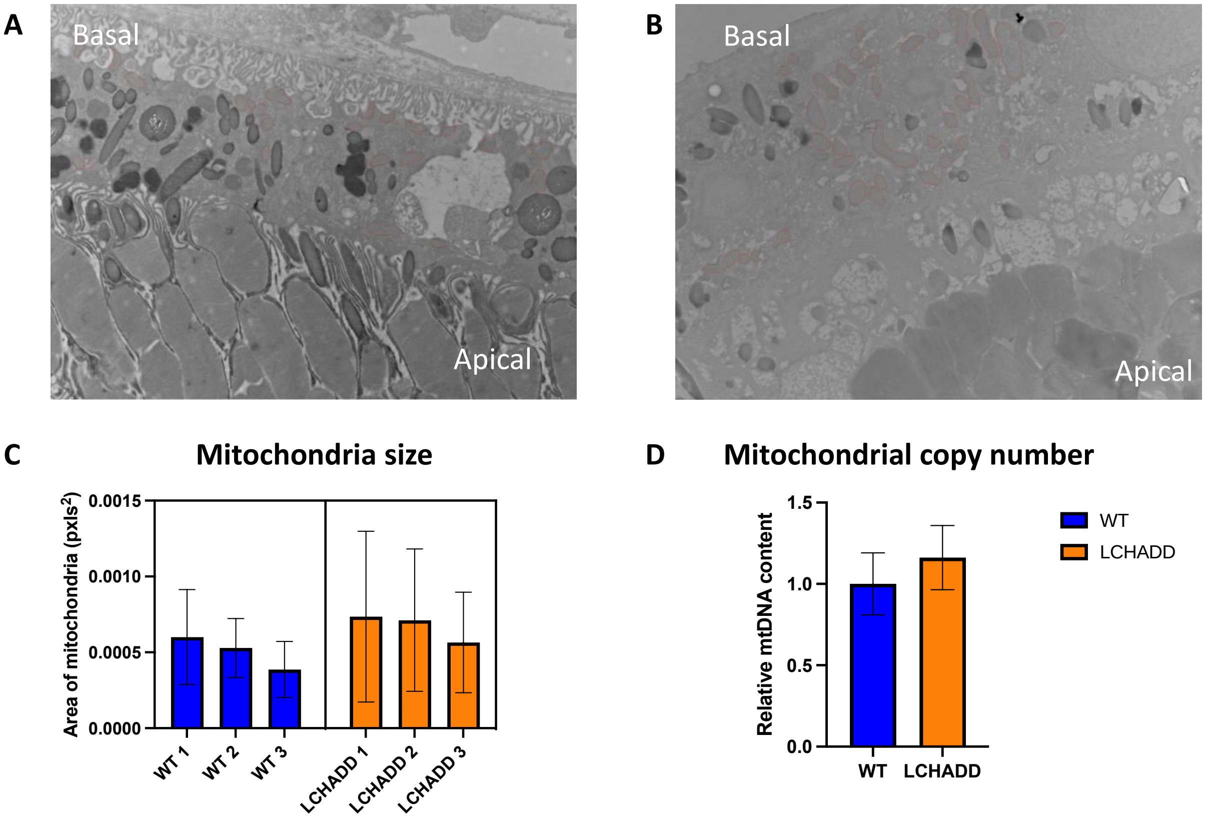

Supplemental Figure 4: LCHADD RPE may have larger mitochondria than WT. (A-B) Representative images of 12-month (A) WT and (B) LCHADD RPE showing that LCHADD RPE may have larger and more abnormally shaped mitochondria (outlined in red). (C) Quantification of the area of mitochondria in WT (n=3 eyes) and LCHADD (n=3 eyes) RPE. While not statistically significant (p=0.11), LCHADD mice trend toward larger mitochondria than WT. Statistics were calculated using a nested t-test. (D) There was no difference in the mitochondrial copy number of 12-month WT (n=4) and LCHADD (n=4) RPE/sclera. Statistics were calculated using a two-tailed T-test.

DEG with -log10(adj p-value)>2		
**Gene**	**log10(Fold change)**	**-log10(adj p-value)**
Pde10a	0.668510184	17.94811901
Egr1	-1.024054017	13.88853934
Egr2	-1.162715397	13.43977612
Arc	-0.933497295	9.892134634
Nr4a1	-0.977054107	9.789660128
Egr3	-1.463945711	8.360534541
Slc25a25	-0.30267469	5.802827659
Mitf	-0.29405198	4.97309374
Myh2	-0.80742496	4.411364701
Errfi1	-0.407837188	3.122906931
Srf	-0.206117621	2.988229353
Dusp1	-0.633690292	2.967839128
Ptgs2	-0.529010862	2.534359789
Dusp8	-0.300325298	2.279426111
Csrp3	-0.657163733	2.242659713
Lrrc30	-0.638548796	2.051352381

Supplemental Table 1: Significant differentially expressed genes (DEGs) in LCHADD mice. Table of all significant DEGs in LCHADD mice. These genes have a -log10(adj p-value)>2 and are all the points highlighted in Figure 6C.
